# Incidence of COVID-19 Among Persons Experiencing Homelessness in the US From January 2020 to November 2021

**DOI:** 10.1001/jamanetworkopen.2022.27248

**Published:** 2022-08-18

**Authors:** Ashley A. Meehan, Isabel Thomas, Libby Horter, Megan Schoonveld, Andrea E. Carmichael, Mitra Kashani, Diana Valencia, Emily Mosites

**Affiliations:** 1 Centers for Disease Control and Prevention, COVID-19 Emergency Response, Atlanta, Georgia; 2 Oak Ridge Institute for Science and Education Fellowship, Oak Ridge Associated Universities, Oak Ridge, Tennessee; 3 Goldbelt C6, LLC, Chesapeake, Virginia

## Abstract

**Importance:**

A lack of timely and high-quality data is an ongoing challenge for public health responses to COVID-19 among people experiencing homelessness (PEH). Little is known about the total number of cases of COVID-19 among PEH.

**Objective:**

To estimate the number of COVID-19 cases among PEH and compare the incidence rate among PEH with that in the general population.

**Design, Setting, and Participants:**

This cross-sectional study used data from a survey distributed by the Centers for Disease Control and Prevention to all US state, district, and territorial health departments that requested aggregated COVID-19 data among PEH from January 1, 2020, to September 30, 2021. Jurisdictions were encouraged to share the survey with local health departments.

**Main Outcomes and Measures:**

The primary study outcome was the number of cases of COVID-19 identified among PEH. COVID-19 cases and incidence rates among PEH were compared with those in the general population in the same geographic areas.

**Results:**

Participants included a population-based sample of all 64 US jurisdictional health departments. Overall, 25 states, districts, and territories completed the survey, among which 18 states (72.0%) and 27 localities reported COVID-19 data among PEH. A total of 26 349 cases of COVID-19 among PEH were reported at the state level and 20 487 at the local level. The annual incidence rate of COVID-19 among PEH at the state level was 567.9 per 10 000 person-years (95% CI, 560.5-575.4 per 10 000 person-years) compared with 715.0 per 10 000 person-years (95% CI, 714.5-715.5 per 10 000 person-years) in the general population. At the local level, the incidence rate of COVID-19 among PEH was 799.2 per 10 000 person-years (95% CI, 765.5-834.0 per 10 000 person-years) vs 812.5 per 10 000 person-years (95% CI, 810.7-814.3 per 10 000 person-years) in the general population.

**Conclusions and Relevance:**

These results provide an estimate of COVID-19 incidence rates among PEH in multiple US jurisdictions; however, a national estimate and the extent of under- or overestimation remain unknown. The findings suggest that opportunities exist for incorporating housing and homelessness status in infectious disease reporting to inform public health decision-making.

## Key Points

**Question:**

How many cases of COVID-19 in the US have occurred among people experiencing homelessness?

**Findings:**

In this cross-sectional study of 64 US jurisdictional health departments, 26 349 cases of COVID-19 among people experiencing homelessness were reported at the state level and 20 487 at the local level. The annual incidence rate of COVID-19 was lower among people experiencing homelessness than in the general population at state and local levels.

**Meaning:**

The findings suggest that incorporating housing and homelessness status in infectious disease surveillance may improve understanding of the burden of infectious diseases among disproportionately affected groups and aid public health decision-making.

## Teaser

This cross-sectional study uses Centers for Disease Control and Prevention data to estimate cases of COVID-19 among people experiencing homelessness at US state and local levels and to compare the incidence rates with those in the general population.

## Introduction

In the US, more than half a million people experience homelessness on any given night.^1^ Compared with the general population, people experiencing homelessness (PEH) have a higher burden of infectious diseases, behavioral health diagnoses, and chronic health conditions.^2,3,4,5^ SARS-CoV-2, the virus that causes COVID-19, is highly transmissible, especially in congregate settings, such as homeless shelters, where there may be crowding and frequent client turnover.^6,7,8^ Between March and September 2020, numerous COVID-19 outbreaks were reported at homeless shelters, but little is known about cases of COVID-19 among PEH in general.^9,10,11,12,13^ Given the infectious disease risks for PEH, it is critical to better understand the true burden of COVID-19 in this population to inform prevention recommendations and provision of care.^14^

The purpose of this study was to gather information from state, territorial, district, county, city, and other local jurisdictions to estimate the number of COVID-19 cases that have occurred among PEH. This study also aimed to compare the incidence rate of COVID-19 among PEH with that in the general population in the jurisdictions collecting these data.

## Methods

### Design, Setting, and Participants

This cross-sectional study was conducted in November 2021. The Centers for Disease Control and Prevention sent state health departments a link to a standardized REDCap survey requesting deidentified and aggregated COVID-19 data among PEH. State health departments were encouraged to share the survey with county and local health departments within their state. Survey questions captured the name and level of the jurisdiction (state, territory, district, county, city, or other); point of contact information for follow-up questions; how the jurisdiction defined homelessness in data collection, including duration of homelessness; the data sources the jurisdiction used to identify or verify housing or homelessness status among COVID-19 cases; the total number of cases of COVID-19 among PEH in their jurisdiction from January 1, 2020, through September 30, 2021; and when available, the proportion of COVID-19 cases among PEH by race and ethnicity and by sex. Responses from states, territories, and districts were grouped and are presented as state- and district-level results, and responses from counties, cities, and other local health agencies were grouped and are presented as local-level results. This research was reviewed by the Centers for Disease Control and Prevention and was conducted consistently with applicable federal law and Centers for Disease Control and Prevention policy (45 CFR §46, 21 CFR §56, 42 USC §241(d), 5 USC §552a, 44 USC §3501, et seq). Written informed consent was obtained from participants at the beginning of the survey. Data are presented descriptively following the Strengthening the Reporting of Observational Studies in Epidemiology (STROBE) reporting guideline.^25^

### Public Data Sources and Measurement of Homelessness

We obtained COVID-19 case data for the general population in the same jurisdictions and during the same time frame as available data on PEH from USAFacts, which aggregates data from the Centers for Disease Control and Prevention and state health departments.^15^ Population size for the respective jurisdictions was obtained from the US Census Bureau.^16^ Publicly available data from the US Department of Housing and Urban Development (HUD) 2020 Point-in-Time (PIT) count served as the population estimates for PEH to calculate the COVID-19 incidence per 10 000 PEH in a subsample of the participating jurisdictions.^1^ The PIT count estimates the number of individuals and families experiencing homelessness during any given night in January of each year and includes counting the number of people living unsheltered (outdoors or in a car, tent, or other place not meant for human habitation) as well as the number of individuals accessing services at emergency homeless shelters or enrolled in transitional housing programs.^17^

### Method for Estimating COVID-19 Incidence Rate Among PEH

Because not all responding jurisdictions collected COVID-19 case data among PEH for the same duration and they used varying definitions of homelessness, we limited the number of jurisdictions used to estimate an incidence rate. Jurisdictions that collected data with at least 1 of the following components in their definition were included in the estimates: person stayed in a homeless shelter, person accessed other homeless services, person slept outside in a place not meant for human habitation, person stayed in transitional housing, person was described as experiencing homelessness in medical records, or person described themselves as experiencing homelessness (such as in a case interview). Jurisdictions that included people staying with friends (“couch surfing”), people staying in permanent supportive housing, and people in hotels or motels were excluded from incidence rate estimates regardless of any other definitions included because their definition of homelessness included experiences not included in HUD’s PIT count, which could lead to an overestimate.

### Statistical Analysis

For jurisdictions that only reported assessing whether a person stayed in a homeless shelter, only the sheltered PIT count was included for the population estimate. Otherwise, the total number of PEH identified in the PIT count was used for the denominator. Free text responses were reviewed to identify whether jurisdictions only selecting “other” for the definition of homelessness should be included in incidence calculations. OpenEpi, version 3.01, was used to calculate incidence rates and 95% CIs.^18^

## Results

### State and District Level Results

Participants included a population-based sample of all 64 US jurisdictional health departments. At the state, territory, and district level, 25 jurisdictions completed the survey, of which 18 (72.0%; 17 states and the District of Columbia) indicated that they collected COVID-19 data among PEH. The District of Columbia and the states that collected data on PEH represent 50.6% of all PEH in the US, indicating that a large number of PEH in the US were not represented in these data.^1^ From January 1, 2020, to November 15, 2021, 17 states and the District of Columbia reported a total of 26 349 cases of COVID-19 among PEH (Table 1). Ten states were included in COVID-19 incidence rate calculations (Table 2). The annual state-level incidence rate of COVID-19 among PEH was 567.9 per 10 000 person-years (95% CI, 560.5-575.4 per 10 000 person-years). The annual incidence rate of COVID-19 in the general population in these same jurisdictions was 715.0 per 10 000 person-years (95% CI, 714.5-715.5 per 10 000 person-years).

**Table 1.** Cases of COVID-19 Among PEH and in the General Population in States and Districts Collecting COVID-19 Data Among PEH From January 2020 to November 2021

Jurisdiction	First date of available data	End date of available data	Duration of data availability, mo.	PEH		General population	
				COVID-19 cases, No.	Estimated No.^a^	COVID-19 cases, No.^b^	Estimated No.^c^
Alaska	Mar 1, 2020	Sep 30, 2021	19.0	1026	2888	108 604	731 545
Arkansas	Apr 1, 2020	Nov 15, 2021	19.5	584	2556	517 193	3 017 804
California	Jan 27, 2020	Sep 30, 2021	20.1	14 903	139 209	4 496 246	39 512 223
Colorado	Jan 1, 2021	Sep 30, 2021	9.0	2364	14 667	337 281	5 758 736
Delaware	Feb 21, 2020	Sep 30, 2021	19.3	345	1825	132 963	973 764
District of Columbia	Apr 1, 2021	Sep 30, 2021	6.0	583	22 351	16 497	705 749
Hawaii	Apr 15, 2021	Sep 30, 2021	5.5	94	6390	48 226	1 415 872
Illinois	Jan 1, 2020	Oct 28, 2021	21.9	1536	24 587	1 693 045	12 671 821
Maine	Jan 1, 2020	Sep 30, 2021	21.0	252	4531	89 989	1 344 212
Minnesota	Apr 5, 2020	Sep 30, 2021	17.8	1134	22 745	710 231	5 639 632
Mississippi	Aug 11, 2020	Sep 29, 2021	13.6	529	2684	419 647	2 976 149
Montana	Jan 1, 2020	Sep 30, 2021	21.0	551	2249	151 033	1 068 778
Oregon	Jan 26, 2020	Sep 30, 2021	20.1	550	17 210	330 055	4 217 737
Pennsylvania	Mar 24, 2020	Sep 30, 2021	18.2	393	8244^d^	1 429 296	12 801 989
Rhode Island	Mar 1, 2020	Sep 30, 2021	19.0	359	3119	172 361	1 059 361
South Dakota	Sep 1, 2020	Sep 30, 2021	13.0	20	1724	131 040	884 659
Tennessee	Apr 6, 2021	Nov 14, 2021	7.3	339	10 519	478 774	6 829 174
Utah	Jan 1, 2020	Sep 30, 2021	21.0	787	6478	508 494	3 205 958
Total	NA	NA	NA	26 349	293 976	11 770 950	103 566 385

Abbreviations: NA, not applicable; PEH, people experiencing homelessness.

^a^ Population estimates of PEH are from the 2020 Point-in-Time estimates reported by the US Department of Housing and Urban Development.^1^

^b^ Cases of COVID-19 in the general population were accessed from USAFacts.^15^

^c^ Population estimates for the entire state are from the US Census Bureau.^16^

^d^ Pennsylvania reported only assessing cases of COVID-19 among PEH who stayed in homeless shelters. The Point-in-Time count includes only people staying in homeless shelters, not the total number of PEH.

**Table 2.** Incidence Rate of COVID-19 Among PEH and in the General Population in Jurisdictions That Collected Data for 12 or More Months and Defined Homelessness Similar to the US Department of Housing and Urban Development Definition From January 2020 to November 2021

Jurisdiction	Duration of follow-up, y	PEH			General population		
		COVID-19 cases, No.	Estimated No.^a^	Incidence rate, per 10 000 person years (95% CI)	COVID-19 cases, No.^b^	Estimated No.^c^	Incidence rate, per 10 000 person-years (95% CI)
**State level**							
California	1.68	14 903	139 209	637.2 (627.1-647.5)	4 496 246	39 512 223	677.3 (676.7-678.0)
Colorado	0.75	2364	14 667	2149.0 (2064.0-2237.0)	337 281	5 758 736	780.9 (778.3-783.6)
Hawaii	0.46	94	6390	319.8 (259.9-389.6)	48 226	1 415 872	740.5 (733.9-747.1)
Illinois	1.83	1536	24 587	341.4 (324.6-358.8)	1 693 045	12 671 821	730.1 (729.0-731.2)
Maine	1.75	252	4531	317.8 (280.4-358.9)	89 989	1 344 212	382.5 (380.0-385.0)
Minnesota	1.48	1134	22 745	336.9 (317.7-356.9)	710 231	5 639 632	850.9 (848.9-852.9)
Oregon	1.68	550	17 210	190.2 (174.8-206.6)	330 055	4 217 737	465.8 (464.2-467.4)
Pennsylvania	1.52	393	8244^d^	313.6 (283.7-345.8)	1 429 296	12 801 989	734.5 (733.3-735.7)
Tennessee	0.61	339	10 519	528.3 (474.3-586.9)	478 774	6 829 174	1149.3 (1146.0-1153.0)
Utah	1.75	787	6478	694.2 (647.0-744.0)	508 494	3 205 958	906.3 (903.8-908.8)
Total	13.51	22 352	254 580	567.9 (560.5-575.4)^e^	10 121 637	93 397 354	715.0 (714.5-715.5)^e^
**Local level**							
Chicago, Illinois (Cook County)	1.54	682	14 433	306.8 (284.5-330.5)	619 536	5 150 233	781.1 (779.2-783.1)
Lexington-Fayette County, Kentucky	1.43	240	1162	1444.3 (1270.0-1636.0)	48 953	323 152	1059.3 (1050.6-1069.2)
San Luis Obispo County, California	1.19	71	228^d^	2616.8 (2059.0-3281.0)	26 199	283 111	777.6 (768.3-787.1)
Stanislaus County, California	1.28	1098	1566	5477.7 (5161.0-5809.0)	71 888	550 660	1671.0 (1659.0-1683.0)
Total	5.44	2091	17 389	799.2 (765.5-834.0)^e^	766 576	6 307 156	812.5 (810.7-814.3)^e^

Abbreviation: PEH, people experiencing homelessness.

^a^ Population estimates of PEH are from the 2020 Point-in-Time estimates reported by the US Department of Housing and Urban Development.^1^

^b^ Cases of COVID-19 in the general population were accessed from USAFacts.^15^

^c^ Population estimates for the entire jurisdiction are from the US Census Bureau.^16^

^d^ Pennsylvania and San Luis Obispo County, California, reported only assessing cases among PEH who stayed in homeless shelters. The Point-in-Time count includes only people staying in homeless shelters, not the total number of PEH.

^e^ The total incidence rate was calculated using the total number of cases divided by the sum of person-time contributed by all included jurisdictions.

Fifteen of the 18 states or districts collecting data among PEH (83.3%) also reported breakdown of cases by race and ethnicity (Table 3). Of the 23 339 cases among PEH at the state and district level with race and ethnicity data, 504 (2.2%) were in American Indian or Alaska Native PEH, 483 (2.1%) in Asian PEH, 4976 (21.3%) in Black PEH, 311 (1.3%) in Native Hawaiian or Pacific Islander PEH, 8087 (34.7%) in White PEH, and 8978 (38.5%) in PEH who reported other race and ethnicity (multiple races and ethnicities or race and ethnicity not already listed). Information on cases among PEH by sex was provided by 16 of the 18 states or districts (88.9%) (Table 3). Of the 25 421 cases with sex information at the state or district level, 12 012 (47.3%) were identified in females. The full gender breakdown of the sample, including nonbinary, agender, gender fluid, or other identity, is not available.

**Table 3.** Cases of COVID-19 Among PEH by Race and Ethnicity and Sex at the State, County, City, or Other Local Level From January 2020 to November 2021

	No. (%)^a^	
	States or districts	Local jurisdictions
**Race and ethnicity** ^b^		
Jurisdictions reporting data	15/18 (83.3)	20/25 (80.0)
COVID-19 cases		
Total	23 339 (100)	15 911 (100)
American Indian or Alaska Native	504 (2.2)	347 (2.2)
Asian	483 (2.1)	393 (2.5)
Black	4976 (21.3)	4783 (30.1)
Native Hawaiian or Pacific Islander	311 (1.3)	179 (1.1)
White	8087 (34.7)	6844 (43.0)
Other^c^	8978 (38.5)	3365 (21.1)
**Sex** ^d^		
Jurisdictions reporting data	16/18 (88.9)	21/25 (84.0)
COVID-19 cases		
Total	25 421 (100)	18 830 (100)
Female^e^	12 012 (47.3)	6211 (33.0)

Abbreviation: PEH, people experiencing homelessness.

^a^ Data were limited to only jurisdictions that collected COVID-19 data among PEH.

^b^ Jurisdictions were asked what percentage of cases among PEH were in each race and ethnicity group. The number of cases by group was calculated using the percentage multiplied by the total number of cases among PEH reported by that jurisdiction.

^c^ Other includes people who identified as more than 1 race and ethnicity or who identifed as a race and ethnicity not otherwise listed.

^d^ Jurisdictions were asked what percentage of cases among PEH were in each sex. The number of cases by sex was calculated using the percentage multiplied by the total number of cases among PEH reported by that jurisdiction.

^e^ Only the number (percentage) of females was reported. The full gender breakdown for jurisdictions, including nonbinary, agender, gender fluid, or other identity, is not available.

The most common components of the definition of homelessness used by the 18 states and districts collecting COVID-19 data among PEH included person stayed in homeless shelter (13 [72.2%]) and person described themselves as experiencing homelessness (13 [72.2%]) (Figure). Sixteen of the 18 states or districts collecting these data (88.9%) considered a person to be experiencing homelessness at the time of the positive COVID-19 test result, and 2 states or districts (11.1%) considered a person to be experiencing homelessness after different durations (1 for at least 12 months and 1 for at least 24 months).

**Figure.**
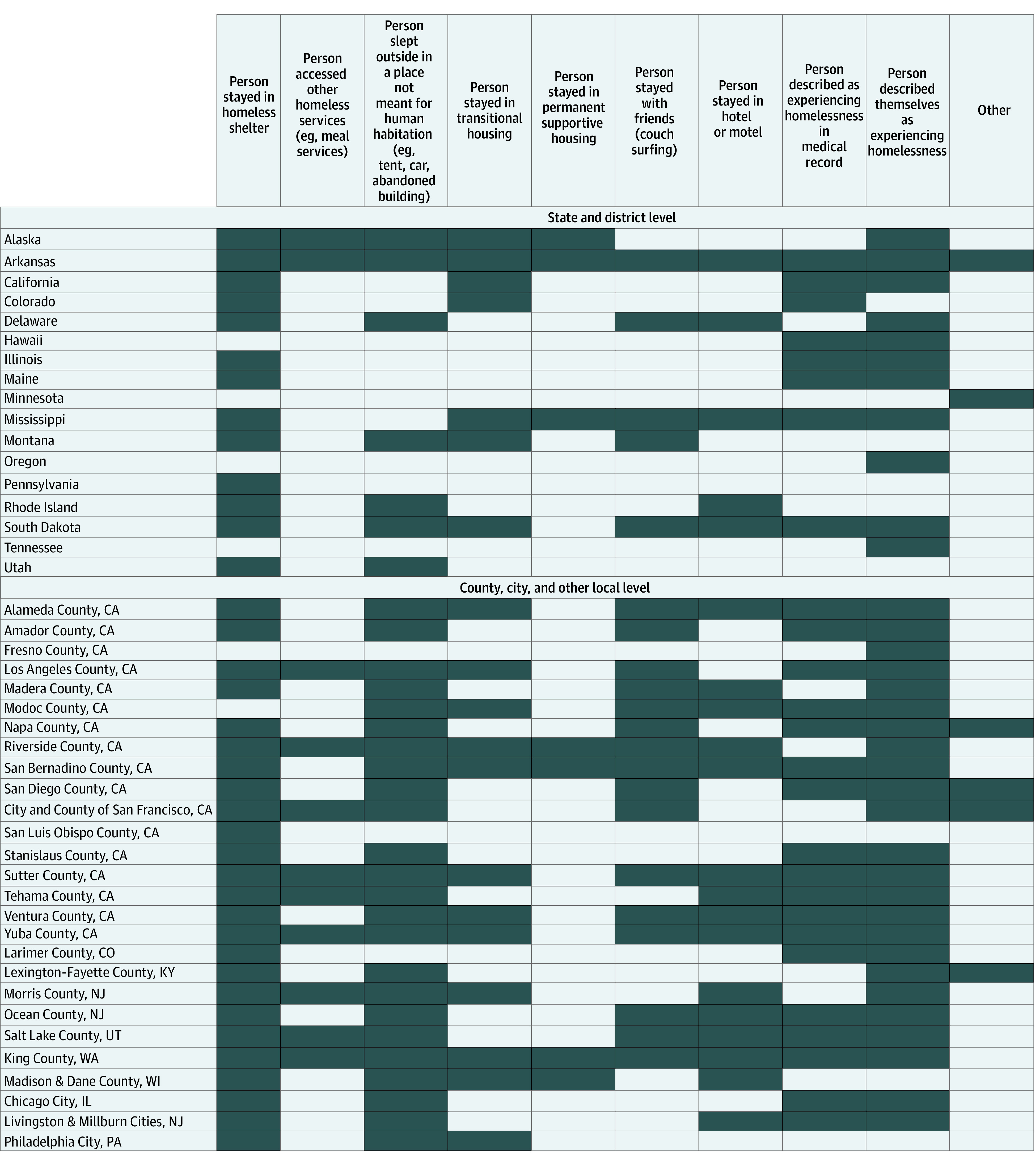
Definitions of Homelessness or Housing Status in State and Local Jurisdictions Collecting COVID-19 Data Among People Experiencing Homelessness, January 2020 to November 2021 Shaded cells indicate factors that the jurisdiction reported using for homelessness in data collection. The District of Columbia collected data among people experiencing homelessness but did not answer how it defined homelessness in data collection.

### County-, City-, and Other Local-Level Results

At the county, city, or other local level, 27 of 39 jurisdictions that completed the survey (69.2%) reported collecting COVID-19 data among PEH. Two pairs of jurisdictions were each combined into a single jurisdiction because they are grouped together in the HUD PIT count, leaving a final sample of 25 local jurisdictions. From January 1, 2020, to November 16, 2021, there were 20 487 cases of COVID-19 reported among PEH at the county, city, or other local level (Table 4). Four localities met the criteria for inclusion in COVID-19 incidence rate calculations (using a definition for homelessness that is consistent with HUD PIT counts) (Table 2). Across these 4 localities, the annual incidence rate of COVID-19 among PEH was 799.2 per 10 000 person-years (95% CI, 765.5-834.0 per 10 000 person-years). The annual incidence rate of COVID-19 in the general population in these same jurisdictions was 812.5 per 10 000 person-years (95% CI, 810.7-814.3 per 10 000 person-years).

**Table 4.** Cases of COVID-19 Among PEH and in the General Population in Counties, Cities, and Other Localities Collecting COVID-19 Data Among PEH From January 2020 to November 2021

Jurisdiction	First date of available data	End date of available data	Duration of data availability, mo	PEH		General population	
				COVID-19 cases, No.	Estimated No.^a^	COVID-19 cases, No.^b^	Estimated No.^c^
California							
Alameda County	Apr 1, 2020	Sep 30, 2021	18.0	993	5853	114 512	1 671 329
Amador County	Jun 12, 2020	Sep 30, 2021	15.6	20	307	5094	39 752
City and County of San Francisco	Mar 20, 2021	Sep 30, 2021	6.3	939	13 389	15 554	881 549
Fresno and Madera Counties^d^	Dec 12, 2020	Sep 30, 2021	9.6	64	2871	69 414	1 156 428
Los Angeles County	Feb 7, 2020	Sep 30, 2021	19.8	9489	46 668	1 394 823	10 039 107
Modoc County	Jul 31, 2020	Nov 16, 2021	15.5	1	792	535	8841
Napa County	Jul 17, 2020	Sep 30, 2021	14.4	62	253	11 602	137 744
Riverside County	Mar 21, 2020	Sep 30, 2021	18.3	725	2347	346 819	2 470 546
San Bernardino County	Mar 11, 2020	Sep 30, 2021	18.6	158	4427	341 118	2 180 085
San Diego County	Jan 1, 2020	Sep 30, 2021	21.0	1442	10 360	356 454	3 338 330
San Luis Obispo County^e^	Jul 22, 2020	Sep 30, 2021	14.3	71	228^e^	26 199	283 111
Stanislaus County	Jun 17, 2020	Sep 30, 2021	15.4	1098	1566	71 888	550 660
Sutter and Yuba Counties^d^	Sep 2, 2020	Sep 30, 2021	12.9	82	417	18 187	175 639
Tehama County	Jul 24, 2021	Sep 23, 2021	2.0	53	273	2013	65 084
Ventura County	Mar 21, 2020	Sep 1, 2021	17.3	120	1845	94 717	846 006
Colorado							
Larimer County	Jan 1, 2020	Sep 30, 2021	21.0	167	1180	35 270	356 899
Illinois							
Chicago^f^	Mar 14, 2020	Sep 30, 2021	18.5	682	14 433	619 536	5 150 233
Kentucky							
Lexington-Fayette County	Apr 24, 2020	Sep 30, 2021	17.2	240	1162	48 953	323 152
New Jersey							
Livingston and Millburn^g^	Apr 30, 2021	Nov 15, 2021	6.5	2	3429	94 174	798 975
Morris County	Apr 16, 2020	Nov 3, 2021	18.6	50	583	54 862	491 845
Ocean County	Dec 26, 2020	Sep 30, 2021	9.1	15	621	59 328	607 186
Pennsylvania							
Philadelphia^h^	Mar 20, 2020	Sep 29, 2021	18.3	316	10 310	174 108	1 584 064
Utah							
Salt Lake County	Mar 19, 2020	Nov 15, 2021	19.9	1144	4769	204 118	1 160 437
Washington							
King County	Mar 11, 2020	Sep 30, 2021	18.6	2392	14 739	151 277	2 252 782
Wisconsin							
Madison and Dane Counties	Apr 4, 2021	Sep 30, 2021	5.9	162	2204	10 723	546 695
Total	NA	NA	NA	20 487	145 026	4 321 278	37 116 479

Abbreviations: NA, not applicable; PEH, people experiencing homelessness.

^a^ Population estimates of PEH are from the 2020 Point-in-Time estimates reported by the US Department of Housing and Urban Development.^1^

^b^ Cases of COVID-19 in the general population were accessed from USAFacts.^15^

^c^ Population estimates for the entire locality are from the US Census Bureau.^16^

^d^ These counties are reported in the same geographic area for population estimates of PEH; thus, they are grouped and presented together.

^e^ San Luis Obispo County, California, reported only assessing cases among PEH who stayed in homeless shelters. The Point-in-Time count includes only people staying in homeless shelters, not the total number of PEH.

^f^ The general population case counts and population size for Chicago, Illinois, are for all of Cook County.

^g^ The general population case counts and population size for Livingston and Millburn, New Jersey, are for all of Essex County.

^h^ The general population case counts and population size for Philadelphia, Pennsylvania, are for all of Philadelphia County.

Twenty of the 25 counties, cities, or other local jurisdictions reporting COVID-19 data among PEH (80.0%) provided race and ethnicity data for cases in this population (Table 3). Of the 15 911 COVID-19 cases among PEH at the county, city, or local level with race and ethnicity data, 347 (2.2%) were in American Indian or Alaska Native PEH, 393 (2.5%) in Asian PEH, 4783 (30.1%) in Black PEH, 179 (1.1%) in Native Hawaiian or Pacific Islander PEH, 6844 (43%) in White PEH, and 3365 (21.1%) in PEH who identified as other race and ethnicity. Twenty-one of the 25 counties, cities, or other local jurisdictions (84.0%) reported the percentage of COVID-19 cases among PEH who were female sex (Table 3). Of the 18 830 cases reported by sex at the local level, 6211 (33.0%) were identified in females. The full gender breakdown of the sample is not available.

The most common components of the definition of homelessness used by the 25 counties, cities, or localities were person stayed in a homeless shelter (25 [100%]), person described themselves as experiencing homelessness (24 [96.0%]), and person slept outside in a place not meant for human habitation (eg, tent, car, abandoned building) (24 [96.0%]) (Figure). Only 1 county, city, or local jurisdiction considered a person to be experiencing homelessness at a duration other than at the time of the positive COVID-19 test, which was experiencing homelessness for at least 1 month.

### Data Sources to Assess Housing or Homelessness Status

Most jurisdictions used multiple data sources to verify a person’s homelessness or housing status. The jurisdictions’ most common data collection method was case investigations or interviews conducted in any modality (eg, in person, by electronic text survey, or by telephone). Reviews of medical records were also used as supplemental information before or after a case investigation or interview and to identify hospitalizations. Many jurisdictions reported that they did not have a process for data matching between the Homeless Management Information System and health data systems.

## Discussion

This cross-sectional study collected the number of COVID-19 cases among PEH and estimated the incidence rate of COVID-19 in multiple jurisdictions. A total of 26 349 COVID-19 cases at the state and district level and 20 487 cases at the local level were reported among PEH. These findings highlight the need for data collection that uses similar definitions of homelessness across data sources. This study adds to the literature by identifying COVID-19 cases over time across multiple jurisdictions.

Considering the risks for other infectious diseases among PEH and the number of disease outbreaks identified in shelters, we expected that the incidence rates of COVID-19 among PEH would be higher than in the general population. The unexpected results found in this study may be explained by a few factors. One is that there could be underascertainment of homelessness among people with COVID-19. For example, community testing that did not collect information on housing status may have excluded some positive results among PEH, leading to underestimated incidence. In addition, because PEH are less likely to seek medical care in general,^19^ they may not have visited health care facilities if they had mild symptoms of COVID-19 and thus may not have been tested. One study in 2021^20^ found that only 59% of individuals with COVID-19 were interviewed for case investigation. For jurisdictions relying on case investigation to determine homelessness status, this lack of interviews could create additional bias.^20^ In addition, data from the present study did not distinguish between sheltered and unsheltered homelessness. Previous data have shown that people experiencing unsheltered homelessness have a lower risk of COVID-19 than do people staying in shelters.^21^ Of note, the composition of PEH in the US is not representative of the general population. Compared with the general population, PEH are more likely to be male and older, and there is disproportionate representation of people who are Black or African American and American Indian or Alaska Native among PEH.^1^ The increased burden of COVID-19 among older adults and racial and ethnic minority individuals across the US is another reason our findings were unexpected.^22,23^

Despite these possible explanations for our findings, given the preventive measures put in place by shelters (eg, physical distancing, ventilation improvements, mask policies, and frequent testing), the results could represent a true balance of risk between PEH and the general population because of these interventions. Of importance, the responding jurisdictions may not be representative of jurisdictions that did not collect data among PEH. Data collection among PEH may indicate high capacity within the health department and could be linked with the ability to support homeless service sites in preventing COVID-19 transmission, or it could indicate that PEH were prioritized for data collection. Considering the data are incomplete and may be biased toward jurisdictions that were well resourced to prevent COVID-19 among PEH, these data should be interpreted with caution.

### Limitations

This study has limitations. Because data could not be collected from all jurisdictions, the number of COVID-19 cases reported among PEH are not an estimate of national incidence. In addition, because of variability in the time frame of available data among PEH, definitions of homelessness, duration of homelessness to be considered homeless, and data sources used to verify housing or homelessness status, COVID-19 estimates could not be compared across jurisdictions. Furthermore, jurisdictions that use more inclusive definitions of homelessness may have different COVID-19 incidence rates than jurisdictions that use narrower definitions of homelessness, contributing to incomparable and possibly skewed estimates. We were not able to explore differences or changes in COVID-19 incidence among PEH during different seasons and in different climates, but it is possible that warmer climates and seasons may be associated with fewer cases of COVID-19 if PEH spend more time outdoors. At the county, city, or local level, COVID-19 incidence rates among PEH may also be affected owing to the reporting of population estimates of PEH from HUD. The number of PEH is reported by HUD at the continuum-of-care level, which does not always directly align with a specific city or county. In rural areas, multiple counties may be grouped together for the population estimate of PEH. Thus, these counties were combined during analysis, but this may have contributed to inaccuracies in incidence rates. There is also a large representation of local jurisdictions from California, and the study team could not control which state-level jurisdictions did or did not share the survey with their local health departments, both of which may bias the local-level results. The few localities used in the incidence calculations included 2 California counties with high rates of COVID-19, which may further bias the local estimates. Of note, there were discrepancies in the number of cases among PEH reported by state and local jurisdictions. For example, Salt Lake City, Utah, reported more cases among PEH than Utah reported for the state overall. This could be explained by differences in definitions for homelessness between Salt Lake City and Utah (Figure) because Salt Lake City had a broader definition of homelessness in data collection. In addition, there may have been variations in testing among PEH across jurisdictions. Some jurisdictional health departments facilitated testing only among people in shelters, and others may have also conducted outreach testing events to people experiencing unsheltered homelessness.

## Conclusions

The results of this cross-sectional study include COVID-19 case counts among PEH in multiple jurisdictions, but a national-level estimate of COVID-19 incidence among PEH and the extent of underestimation or overestimation in these results remains unknown. Data on infection incidence rates during public health emergencies could be used to inform policy decisions and resource allocation to reduce the burden of infectious diseases among PEH. Possible opportunities for public health practice include integration of homeless service utilization data systems, such as the Homeless Management Information System, into health data systems.^24^ Integration of these data systems may alleviate burden on health departments in collecting housing or homelessness information during case investigations or interviews and would further support ongoing data modernization initiatives. In addition, health departments could consider creating public-facing dashboards or regularly posted reports with these data, which would allow for improved data sharing and informed decision-making at all levels of public health responses. Opportunities exist for incorporating housing and homelessness status in infectious disease reporting to inform public health actions.

## References

[1] US Department of Housing and Urban Development. 2020 AHAR: Part 1—PIT estimates of homelessness in the US. Published March 2021. Accessed October 18, 2021. https://www.huduser.gov/portal/datasets/ahar/2020-ahar-part-1-pit-estimates-of-homelessness-in-the-us.html

[2] Baggett TP, Liauw SS, Hwang SW. Cardiovascular disease and homelessness. J Am Coll Cardiol. 2018;71(22):2585-2597. doi:10.1016/j.jacc.2018.02.077 29852981

[3] Bartels SJ, Baggett TP, Freudenreich O, Bird BL. COVID-19 emergency reforms in Massachusetts to support behavioral health care and reduce mortality of people with serious mental illness. Psychiatr Serv. 2020;71(10):1078-1081. doi:10.1176/appi.ps.202000244 32487009

[4] Kuhn R, Richards J, Roth S, Clair K. Homelessness and public health in Los Angeles. March 31, 2020. Accessed October 28, 2021. https://escholarship.org/uc/item/2gn3x56s

[5] Snyder LD, Eisner MD. Obstructive lung disease among the urban homeless. Chest. 2004;125(5):1719-1725. doi:10.1378/chest.125.5.1719 15136382

[6] Ghinai I, Davis ES, Mayer S, . Risk factors for severe acute respiratory syndrome coronavirus 2 infection in homeless shelters in Chicago, Illinois—March-May, 2020. Open Forum Infect Dis. 2020;7(11):ofaa477. doi:10.1093/ofid/ofaa477 33263069 PMC7665740

[7] Rogers JH, Link AC, McCulloch D, ; Seattle Flu Study Investigators. Characteristics of COVID-19 in homeless shelters: a community-based surveillance study. Ann Intern Med. 2021;174(1):42-49. doi:10.7326/M20-3799 32931328 PMC7517131

[8] Sanche S, Lin YT, Xu C, Romero-Severson E, Hengartner N, Ke R. High contagiousness and rapid spread of severe acute respiratory syndrome coronavirus 2. Emerg Infect Dis. 2020;26(7):1470-1477. doi:10.3201/eid2607.200282 32255761 PMC7323562

[9] Baggett TP, Keyes H, Sporn N, Gaeta JM. Prevalence of SARS-CoV-2 infection in residents of a large homeless shelter in Boston. JAMA. 2020;323(21):2191-2192. doi:10.1001/jama.2020.6887 32338732 PMC7186911

[10] Imbert E, Kinley PM, Scarborough A, . Coronavirus disease 2019 outbreak in a San Francisco homeless shelter. Clin Infect Dis. 2021;73(2):324-327. doi:10.1093/cid/ciaa1071 32744615 PMC7454344

[11] Mohsenpour A, Bozorgmehr K, Rohleder S, Stratil J, Costa D. SARS-Cov-2 prevalence, transmission, health-related outcomes and control strategies in homeless shelters: systematic review and meta-analysis. EClinicalMedicine. 2021;38:101032. doi:10.1016/j.eclinm.2021.101032 34316550 PMC8298932

[12] Mosites E, Parker EM, Clarke KEN, ; COVID-19 Homelessness Team. Assessment of SARS-CoV-2 infection prevalence in homeless shelters—four US cities, March 27–April 15, 2020. MMWR Morb Mortal Wkly Rep. 2020;69(17):521-522. doi:10.15585/mmwr.mm6917e1 32352957 PMC7206983

[13] Tobolowsky FA, Gonzales E, Self JL, . COVID-19 outbreak among three affiliated homeless service sites—King County, Washington, 2020. MMWR Morb Mortal Wkly Rep. 2020;69(17):523-526. doi:10.15585/mmwr.mm6917e2 32352954 PMC7206987

[14] Mosites E, Harrison B, Montgomery MP, . Public health lessons learned in responding to COVID-19 among people experiencing homelessness in the United States. *Public Health Rep*. Published online April 29, 2022. doi:10.1177/00333549221083643 PMC9066269 35485305

[15] USAFacts. US COVID-19 cases and deaths by state. Accessed October 28, 2021. https://usafacts.org/visualizations/coronavirus-covid-19-spread-map

[16] US Census Bureau. County population totals: 2010-2019. Accessed October 18, 2021. https://www.census.gov/data/datasets/time-series/demo/popest/2010s-counties-total.html

[17] US Department of Housing and Urban Development Exchange. HDX FAQs: FAQ ID 1818. Published December 2014. Accessed October 28, 2021. https://www.hudexchange.info/faqs/reporting-systems/homelessness-data-exchange-hdx/pit/pit-general/for-purposes-of-the-point-in-time-pit-count-who-does-hud-consider/#:~:text=HUD's%20PIT%20count%20is%20limited,the%20time%20of%20the%20count

[18] Dean AG, Sullivan KM, Soe MM. OpenEpi: open source epidemiologic statistics for public health. Updated April 6, 2013. Accessed June 28, 2022. http://www.OpenEpi.com

[19] Moore G, Gerdtz M, Manias E, Hepworth G, Dent A. Socio-demographic and clinical characteristics of re-presentation to an Australian inner-city emergency department: implications for service delivery. BMC Public Health. 2007;7:320. doi:10.1186/1471-2458-7-320 17996112 PMC2222161

[20] Lash RR, Moonan PK, Byers BL, ; COVID-19 Contact Tracing Assessment Team. COVID-19 case investigation and contact tracing in the US, 2020. JAMA Netw Open. 2021;4(6):e2115850. doi:10.1001/jamanetworkopen.2021.15850 34081135 PMC8176334

[21] Yoon JC, Montgomery MP, Buff AM, . Coronavirus disease 2019 (COVID-19) prevalences among people experiencing homelessness and homelessness service staff during early community transmission in Atlanta, Georgia, April-May 2020. Clin Infect Dis. 2021;73(9):e2978-e2984. doi:10.1093/cid/ciaa1340 32898272 PMC7499502

[22] Hsu HE, Ashe EM, Silverstein M, . Race/ethnicity, underlying medical conditions, homelessness, and hospitalization status of adult patients with COVID-19 at an urban safety-net medical center—Boston, Massachusetts, 2020. MMWR Morb Mortal Wkly Rep. 2020;69(27):864-869. doi:10.15585/mmwr.mm6927a3 32644981 PMC7727597

[23] Rossen LM, Branum AM, Ahmad FB, Sutton P, Anderson RN. Excess deaths associated with COVID-19, by age and race and ethnicity—United States, January 26-October 3, 2020. MMWR Morb Mortal Wkly Rep. 2020;69(42):1522-1527. doi:10.15585/mmwr.mm6942e2 33090978 PMC7583499

[24] US Department of Housing and Urban Development Exchange. Homeless management information system. Accessed October 18, 2021. https://www.hudexchange.info/programs/hmis/

[25] von Elm E, Altman DG, Egger M, Pocock SJ, Gøtzsche PC, Vandenbroucke JP; STROBE Initiative. The Strengthening the Reporting of Observational Studies in Epidemiology (STROBE) statement: guidelines for reporting observational studies. J Clin Epidemiol. 2008;61(4):344-349. doi:10.1016/j.jclinepi.2007.11.008 18313558

